# Study on characters associations and path coefficient analysis for quantitative traits of amaranth genotypes from Ethiopia

**DOI:** 10.1038/s41598-023-47869-0

**Published:** 2023-11-28

**Authors:** Mekonnen Yeshitila, Andargachew Gedebo, Hewan Demissie Degu, Temesgen Magule Olango, Bizuayehu Tesfaye

**Affiliations:** 1 Dilla College of Education, P. O. Box 334, Dilla, Ethiopia; 2 School of Plant and Horticultural Sciences, Hawassa University, Hawassa, Ethiopia

**Keywords:** Biomaterials, Ecology, Plant sciences

## Abstract

Selection based on yield alone may not be effective for yield improvement in plant breeding programs. Thus, in order to progress the genetic gains during selection, yield should be considered along with potential yield contributing traits. The objective of this study was to improve the genotype of amaranth and increase the effectiveness of selection in the program by identifying the correlation and path coefficients between yield and its relevant attributes. On 120 genotypes of amaranth planted during two growing seasons in 2020 and 2021, the study was carried out using an alpha lattice design with two replications. The results revealed significant positive phenotypic and genotypic associations on leaf yield, with leaf area, leaf breadth, branch number, leaf number, plant height at flowering, and grain yield all having positive direct effects. Similar strong positive phenotypic and genotypic relationships were found for grain yield and grain sink filling rates. Using path coefficient analysis, the direct and indirect effects of yield-related traits on yield were also determined. In addition to having a strong direct impact on grain output, the grain sink filling rates showed both phenotypic and genotypic evidence of substantial positive relationships with grain yield. It was further suggested that leaf yield in amaranth genotypes may increase through the indirect selection of plant height at maturity, leaf length, and terminal inflorescence lateral length, which showed such significant indirect influences, mostly through leaf area, days to maturity, and days to emergence, which displayed such strong indirect effects, primarily through plant height at flowering. This study consequently shows the need for traits with significant positive indirect impacts via leaf area to be considered indirect selection criteria for improving leaf yield in amaranth genotypes. The grain sink filling rate also significantly improved grain yield indirectly at both the phenotypic and genotypic levels, mainly via days to flowering and leaf yield. This demonstrated that selection that mainly targeted days to flowering, leaf yield, and grain sink filling rate would ultimately boost the grain yield in amaranth genotypes.

## Introduction

A diversified family of food crops known as amaranths is impressively adaptable to new environments despite a variety of biotic and abiotic obstacles^1^. Practically speaking, it is a pseudo-cereal because it is dicotyledonous and not a true cereal^2^. There are 87 species in the genus^3^. However, because of the paucity of systematic studies on Amaranths, the number of species is unclear, and the genus is regarded as challenging by systematics^4^.

Amaranths are now becoming more significant in both human and animal nourishment^5^. They have a balanced amino acid profile and high seed protein concentration^6^. Amaranth is high in iron, calcium, potassium, phosphorus, protein, and lysine. It also contains vitamins A and C^7^. The Food and Agriculture Organization of the United Nations views them as a critical crop for food and nutritional security because of their nutritional value (FAO)^8^. Amaranths are a great substitute for cereal for those with celiac disease due to their low gluten level^9^.

The complex variable of yield is influenced by many different variables, notably polygenes, the environment, and genetic variability^10,11^. Understanding the relationships between quantitatively inherited crop traits and their direct and indirect effects on yield is crucial for the success of breeding program selections^12^. Moreover, studies on the genotypic and phenotypic correlations among crop plant traits are useful for planning, assessing, and creating selection criteria for the targeted characters for selection in the breeding program^13^. Generally speaking, the strength of character association has a significant impact on the effectiveness of any crop development effort.

Path coefficient analysis is an efficient statistical technique specially designed to quantify the interrelationship of different components and their direct and indirect effects on yield. Partitioning of total correlation into direct and indirect effects by path coefficient analysis helps in making the selection more effective^14^. Keeping these facts under consideration, a planned effort was undertaken to evaluate different genotypes of amaranth under the agro-climatic conditions of Ethiopia. The selection of better genotypes for breeding programs depends on this information. There has not been a remarkable study conducted in Ethiopia to date that evaluates the relationships between yield components and plant characteristics and grain and leaf yield, as well as the direct and indirect effects of these relationships on grain and leaf yield in different amaranth genotypes. To obtain the yield data required for amaranth breeding and development, the objective of this study was to (1) quantify the magnitude of the correlation coefficient and path coefficients between traits of amaranth genotypes grown in Ethiopia. (2) Analyse the direct and indirect effects of quantitative features on grain and leaf yields. (3) Put out the selection criteria for boosting amaranth genotypes' grain and leaf yields.

## Material and methods

### Experimental Site

**Figure 1:**
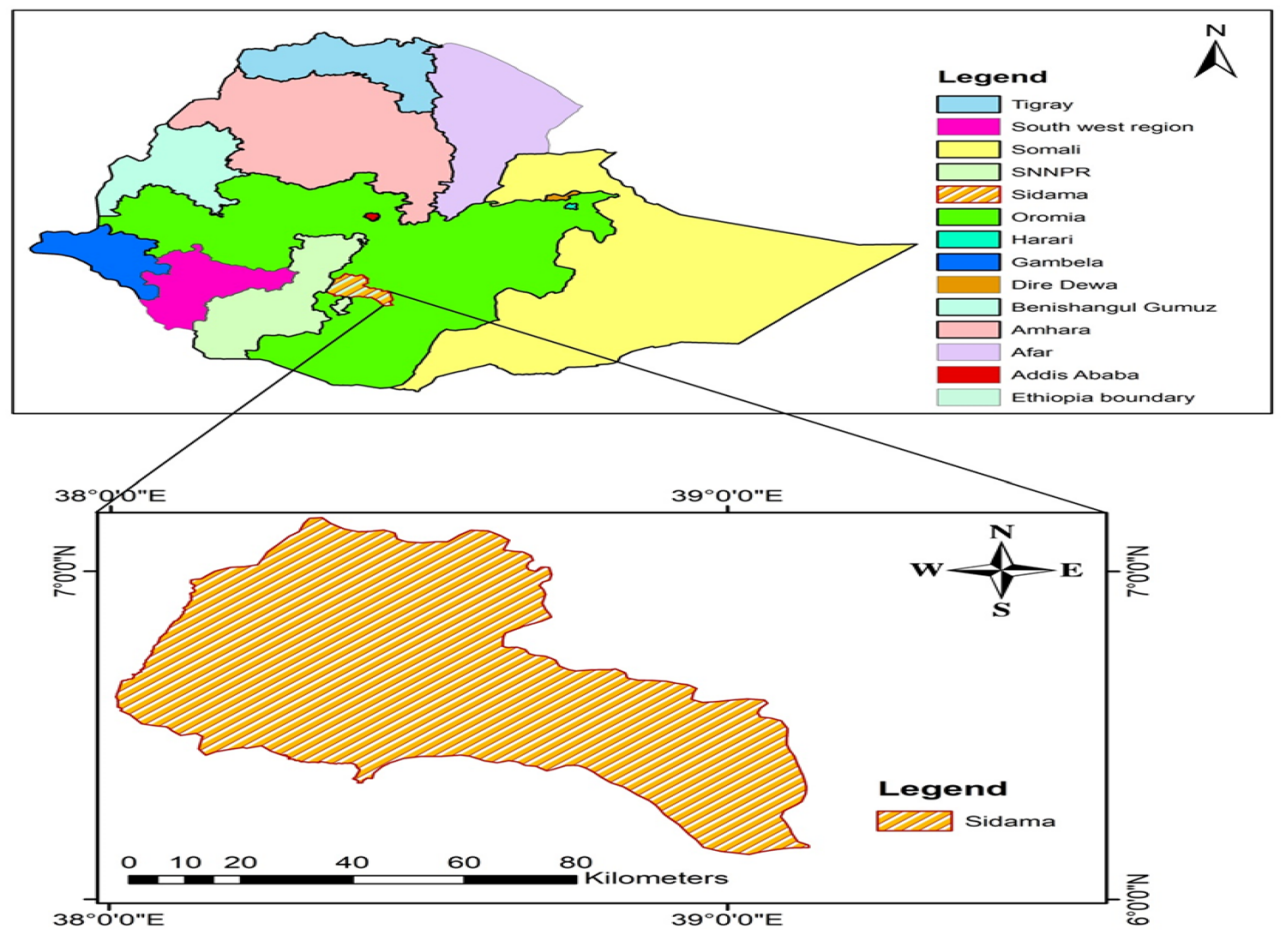
Map of the study area. Maps were generated using Arcmap v 10.6 1.

The experiment was conducted in 2020 and 2021 at the agricultural experimental site, Hawassa University. It is geographically located around 275 kms from Ethiopia's capital, Addis Ababa, in the Sidama Region. The experimental area is situated at an altitude of 1709 m above sea level, at latitude 7°2′ 54.7503′′ N and longitude 38°30′ 17.1608′′ E (Fig. 1). Clay loam was the dominant soil texture class in the experimental location, where the pH ranged from 6 to 6.5. The district's mean monthly low and high temperatures are, respectively, 14.10 °C and 27.9 °C. In all, throughout the two growing seasons, the experimental farm received 1379.16 mm of rain.

### Plant material

Amaranth was collected legitimately and under the proper national (Ethiopian Biodiversity Institute) and international guidelines. The Ethiopian Biodiversity Institute field staff assisted in collecting the samples that were used as vouchers. The specimens were accepted by the National Herbarium (ETH) at Addis Abeba University after they had been placed there, dried, tagged, pressed, and given a number. The specimens were identified by systematist Melaku Wondafrash Ayitaged at the Addis Abeba University National Herbarium.

A total of 120 amaranth genotypes were used in this study, with 34 genotypes coming from the Ethiopian Biodiversity Institute (EBI), 2 from the Melkasa Research Center, 15 from the Werere Research Center (Afar region), 8 from Sidama, and the remaining 61 genotypes being collected in the Southern Nations, Nationalities and Peoples Region, Oromia, and Tigray regions of Ethiopia in 2019 and characterized for various agro-morphological traits. One hundred eighteen members contain passport information, but the other two do not and are regarded as released varieties (Table 1).

**Table 1:** List and passport data of plant materials included in the study.

Genotype name	Easting	Northing	Altitude
KEN-065	321,813	583,339	1756
YRC-048	411,783	675,273	1995
KAZ-077	327,268	599,608	1202
KEN-067	321,745	585,148	1760
211457	326,440.36	589,244.33	1560
242917	812,954	793,136	2200
KEN-011	299,762	603,070	1739
241764	306,077	591,230	1520
HA-003	636,126	1,046,188	726
KAZ-076	327,267	599,336	1202
208683	758,052	1,043,629	2270
KEN-016	322,071	584,756	1745
MEK-084	552,161	1,490,423	2155
HAL-039	407,697	806,621	1834
212892	315,406	622,936	2200
211455	328,390	645,017	1150
HAW-041	444,076	776,871	1750
KAZ-055	326,719	609,804	1213
DRA-053	324,631	614,748	1290
ALE-023	299,964	597,729	1370
KEN-015	320,119	586,057	1819
ALE-069	299,971	597,724	1376
DIL-050	421,120	709,519	1459
KAZ-057	326,722	609,738	1214
KEN-022	321,811	585,162	1751
SHIA-007	627,807	1,036,846	735
240815	354,053.54	754,795.3	1950
209056	312,304.71	699,421.8	1660
ABL-004	369,796	734,542	1367
ALE-034	295,826	597,501	1316
ALE-024	299,885	597,965	1368
SAA-004	636,126	1,046,188	726
212582	572,790	1,234,490	1840
ALE-068	299,970	597,736	1384
212581	581,861	1,245,570	2920
KAZ-007	327,352	599,054	1187
204645	327,492.26	590,316.19	1600
225717	348,942	740,795	1440
KEN-019	321,816	585,168	1754
91003	233,325	835,289	2040
242533	665,945	1,167,179	1250
KEN-013	319,707	586,409	1772
BNT-026	247,228	615,060	1618
AC-NL	Released variety	Released variety	1550–2400
Madiira II	Released variety	Released variety	1550–2400
BKD-027	232,336	637,249	1427
204644	334,751.47	653,902.46	1200
225718	348,942	740,795	1440
ALE-070	299,981	597,743	1329
OTI-044	431,913	721,434	1893
KAZ-008	326,435	604,708	1204
MEK-081	552,793	1,489,902	2143
KAZ-056	326,684	609,708	1214
KEN-018	321,785	585,144	1761
BA-001	628,326	1,026,376	743
ALE-074	299,762	603,000	1655
209057	341,220.34	740,810	1580
KAZ-059	327,268	599,608	1202
KEN-010	319,325	585,745	1729
KEN-012	319,703	586,470	1760
215567	372,960	768,376	2100
ALE-025	300,048	597,628	1349
SHD-001	434,789	761,187	1874
ALE-073	301,337	604,237	1693
DAL-043	429,799	738,442	1790
SA-001/07	624,955	1,028,024	736
SRA-002	632,177	1,031,734	742
219284	358,268	783,157	1750
HA-008	634,804	1,044,976	730
ALE-071	299,973	597,729	1372
240814	322,866.04	764,187.65	1250
KAZ-060	319,338	585,751	1734
MEK-083	552,491	1,490,761	2151
BKD-031	234,460	634,949	1400
WON-049	418,632	699,405	1743
KAZ-058	327,268	599,608	1202
KAZ-006	327,308	598,524	1188
BKD-029	234,471	634,953	1409
WA-003	628,204	1,033,787	741
YRB-002	424,075	769,481	1857
KEN-021	321,818	585,166	1752
KAZ-079	326,433	604,711	1204
KEN-064	321,816	585,168	1762
SHL-040	433,633	805,425	1662
KEN-017	322,084	584,791	1762
242532	665,945	1,167,179	1250
SA-008/07	624,844	1,026,573	740
240813	323,825.31	589,809.06	2750
208764	698,483.15	943,746.8	1850
MEK-080	552,552	1,490,147	2162
BA-016	629,359	1,027,955	740
WA-001	627,878	1,033,651	740
SA-025/07	625,039	1,026,746	735
DMG-038	380,667	777,365	1881
KAZ-078	327,269	599,337	1200
242531	195,529	1,185,259	1250
DA-006	630,310	1,034,519	735
211456	314,087.91	599,272.76	1570
CHU-045	431,835	721,533	1910
DUF-003	394,497	770,836	2113
ADZ-037	389,243	797,508	1912
212583	570,917	1,258,445	1640
208025	300,580	1,354,876	2100
225713	311,940	696,674	1600
KEN-014	319,660	586,850	1690
91001	315,299	584,231	1890
212893	324,653	628,440	1380
ALE-033	282,131	591,864	589.5
KEN-020	321,817	585,166	1753
K3A-005	631,670	1,035,541	738
242530	665,945	1,167,179	1250
BKD-028	234,471	634,953	1409
SA-005/07	624,847	1,026,721	737
KAZ-009	325,782	604,450	1213
242534	665,945	1,167,179	1250
212890	382,230	795,996	2180
DAL-042	431,596	753,041	1780
MEK-082	552,972	1,490,517	2140
225715	258,450	696,873	1780
ALE-075	299,919	603,075	1640

### Experimental layout

A tractor cleared the experimental field, correctly plowed it, and harrowed it. A manual hoe was used to prepare the ridges. The experimental design was an alpha lattice. A layout with 2 replications, 16 blocks, and 15 plots per block for amaranths was conducted. Each unit plot was separated by a 0.60-m distance between plots, a one-meter distance between blocks, and a three-meter distance between replications, with a plot size of 1.80 m in length and 1.50 m in width for a 2.7 m^2^ area. Each season required a total space of 1345.2 m^2^ (38 m × 35.4 m). On April 15th, 2020, and 2021, the seed was sown at one location in each season, which is under ideal growing circumstances and during the agricultural season. One growing season on the experimental site in one year is considered the environment. Seeds of various genotypes (Table 1) were consistently sowed in two rows with a gap of 0.75 m between them. Seeds are quite tiny, with sizes ranging from 0.37 to 1.21 g per 1000 seed weight^15^, and they were planted in seedbeds and covered with powdered, finely diced cow farmyard manure after being combined with sand in a 1:4 ratio. At 14 and 22 days after sowing (DAS), thinning was done twice at a distance of 75 cm between rows and 30 cm between plants. According to Grubben and Van Sloten^16^ and Shukla, et al.^17^, the experiment followed standard cultural practices. Hand-hoeing was used to control weeds at 2-week intervals following germination and whenever necessary. A total of 12 plants were maintained in each plot.

### Data collection

Data on agro-morphological traits were collected either on a plot-by-plot basis or from ten randomly selected plants per plot. A list of the studied 24 agro-morphological traits with their descriptions and sampling methods is indicated in Table 2. At various phonological phases, observations were conducted on a variety of morphological characteristics. Ten randomly selected plants in each plot had their phenotypic traits evaluated. The International Board for Plant Genetic Resources suggested utilizing amaranth descriptors to describe mature plants based on taxonomic keys^18^. Characters that were not on the list but were deemed crucial for the characterization were included. To accomplish the aforementioned objectives, a two-season experiment was carried out at the Agricultural Research Site of Hawassa University, Ethiopia.

**Table 2:** List of quantitative agro-morphological traits used along with their marker code, unit, basis of sampling, and description.

Phenotypic markers	Marker code	Unit	Basis of sampling	Descriptions
Stem diameter	SD	cm	Plant	Measured thickest part (10 cm) above ground of the stem at the lower part at the maturity stage by using a digital caliper
Leaf thickness	LT	mm	Plant	Three different representative leaf sizes (small, middle, and large) per plant were randomly sampled for measurement of leaf thickness in the middle of the leaf at the flowering stage by using a digital caliper
Grain sink filling rate	GSFR	kg ha^−1^ day^−1^	Plot	GSFR is stated as a ratio of grain yield to the number of days from 50% blooming to seed maturity
Leaf width	LW	cm	Plant	Three different representative leaf sizes (small, middle, and large) per plant were randomly sampled for measurement of leaf width at the flowering stage by using a meter tap
Plant height at flowering	PHF	cm	Plant	Measured from the ground surface to the tip of the main stem in cm at the 50% flowering stage by using a meter tap
Plant height at maturity	PHM	cm	Plant	Measurement of the height of a plant from the soil surface to the top of the inflorescence at the 80% maturity stage by using a meter tap
Petiole length	PL	cm	Plant	Three different representative petiole sizes (small, middle, and large) per plant were measured in centimeters from the base of the stem to the petiole of the leaf with the help of a meter scale at the flowering stage
Branch number	BN	Number	Plant	The number of branches was counted along the main stem at the maturity growth stage
Terminal inflorescence stalk length	TISL	cm	Plant	The mean length of 10 randomly selected terminal inflorescence stalks was measured with a meter rule starting from the base up to the tip of the inflorescence at the maturity stage
Terminal inflorescence laterals length	TILL	cm	Plant	The mean length of 10 randomly selected terminal inflorescence laterals length (cm) was measured at the maturity stage
Number of nodes on the main stem	NN	Number	Plant	Many nodes were counted along the main stem at the flowering stage
Grain yield	GY	tone ha^−1^	Plot	Grain yield was estimated at an adjusted moisture content of 12% and weighed to determine the dry matter of the net plot and then extrapolated per hectare
Leaf area	LA	cm^2^	Plant	Three different representative leaf sizes (small, middle, and large) per plant were measured by using a digital leaf area meter
1000 seed weight	TSW	g	Plot	A Sample of thousand seeds from each genotype was counted and weighed using an analytical balance
Leaf number	LN	Number	Plant	The main countable leaves were counted along the main stem at the flowering stage
Days to maturity	DM	Days	Plot	The number of days between 90% emergence and the physiological maturity of 80% of plants. (The time of plant maturity is when the seed taken from the central part of the inflorescence does not change shape when pressed between fingers and when the inflorescence changes its color from green to brown)
Days to flowering	DF	Days	Plot	From the 90 percent emergence date to the stage where ears had appeared in the terminal inflorescence from 50 percent of the plants in a row, days to 50 percent bloom
Basal lateral branch length at maturity	BLBL	cm	Plant	Measured from the base of the stem branches to the top of a branch at the maturity stage by using a meter tap
Top lateral branch length at maturity	TLBL	cm	Plant	Measured from the top of the stem branches to the top of a branch at the maturity stage by using a meter tap
Axillary inflorescence length	AIL	cm	Plant	The mean length of ten randomly selected auxiliary inflorescence was measured at the maturity stage
Days to emergence	DE	Days	Plot	The number of days from planting to 90% of seedlings germinates in each plot was recorded and expressed in days
Leaf yield	LY	tone ha^−1^	Plot	The separated parts of the leaves were dried for a week (7 days) and weighed to determine the dry matter of the net plot and then extrapolated per hectare
Grain filling period	GFP	Days	Plot	The number of days between days for 50% flowering and days to grain maturity
Leaf length	LL	cm	Plant	Three different representative leaf sizes (small, middle, and large) per plant were randomly sampled for measurement of leaf length at the flowering stage by using a meter tap

### Statistical analysis

#### Diagnosis of multicollinearity

One of the requirements for accurate and trustworthy path coefficient estimation is the analysis of multicollinearity between any explanatory variables^19^. The key plant breeding phenomena of collinearity diagnostic boosts the effectiveness of indirect selection for genetic improvement when the target characteristics have low heritability^20^. When there is a moderate to severe multicollinearity, the computed coefficients may have utterly irrational (higher than unity) values, and the variances linked to estimators of path coefficients may inflate, generating incorrect estimates^21^, and the variances associated with estimators of path coefficients may become inflated and leads to unreliable estimates^22^. By examining the correlation matrix or by using the variance inflation factor (VIF), tolerance limit (TOL), and eigenvalues, multicollinearity can be identified. Two explanatory variables may be multicollinear if there are strong coefficients (near + 1) between them in the correlation matrix^23^. This possibility can be verified using the VIF, TOL, and eigenvalues. Using the VIF, TOL, and COLLIN option methods, the PROC REG was used to perform the VIF, TOL, and eigenvalues (SAS).

A number greater than 10 indicates that the related regression coefficients are underestimated due to multicollinearity^24^. The VIF quantifies how much the variance of an estimated regression coefficient is increased due to the effect of multicollinearity^25^. The TOL, which is the inverse of the VIF, indicates that there is severe collinearity in the database when it is larger than 0.1^25^. According to Montgomery et al.^25^, the correlation matrices' maximum eigenvalue (max) can be divided by the subsequent smallest eigenvalue (min) to determine the condition number (CN) for each trait. Montgomery et al.^25^ determined that the degree of multicollinearity in the matrices was categorized as weak when the estimates of CN were less than 100, moderate when they were between 100 and 1000, and severe when they were greater than 1000. Either eigenvalues or eigenvectors can be used simultaneously to find the multicollinearity issue. Therefore, removing the traits responsible for boosting the variance of the regression coefficient is essential unless the trait is of importance for improvement. Traits with a low eigenvalue and a high matching eigenvector indicate a striking collinearity problem^22^. Therefore, removing the traits that cause multicollinearity from the study is a quicker and more efficient way to solve this issue and enables obtaining the most precise path coefficients^23^. As a result, the correlation matrices, VIF, TOL, CN, and Eigen system analysis of the correlation matrix were used to perform the multicollinearity test between the researched features. In total 24 traits were measured and based on the multicollinearity diagnosis tests. These multicollinearity diagnosis tests allowed us to exclude variables like TSW, GFP, BLBL, TLBL, SD, NN, PL, and LT from the analysis of correlation since they were so strongly linked to multicollinearity in the matrix. However, multicollinearity is associated with DM according to grain yield path analysis and GSFR related to leaf yield path analysis; these features were removed as well from the analysis. The remaining 16 variables (or around 66%) and 14 variables (58.33%) were considered for correlation and path, respectively, for the analysis due to their low multicollinearity in the current study.

### Phenotypic and genotypic correlations

The collected data from the two years were combined and subjected to correlation and path coefficients analysis. Correlation analysis was performed for all possible combinations of traits after diagnosing the multicollinearity test. Phenotypic correlation, the observable correlation between two explanatory traits, which includes both genotypic and environmental effects, and genotypic correlation, the inherent association between two explanatory traits, were computed from the components of variance and co-variances as described by^26^. The coefficient of correlation was tested using ‘r’ tabulated value at n − 2 degrees of freedom, at 5, 1, and 0.1% probability level, where n is the number of genotypes.

1 $${\mathrm{rp}}_{\rm{XY}}=\frac{{\mathrm{PCOV}}_{(\mathrm{x },\mathrm{y})}}{\sqrt{{{\upsigma }^{2}}_{{\mathrm{P}}_{\rm{x}}}* {\upsigma }^{2}{\mathrm{P}}_{\rm{y}}}}$$

2 $${\mathrm{rg}}_{\rm{XY}}=\frac{{\mathrm{GCOV}}_{(\mathrm{x },\mathrm{y})}}{\sqrt{{{\upsigma }^{2}}_{{\mathrm{g}}_{\rm{x}}}* {\upsigma }^{2}{\mathrm{g}}_{\rm{y}}}}$$

where rp_xy_ and rg_xy_ are the phenotypic and genotypic correlation coefficient between traits x and y, Pcov(x,y) and Gcov(x,y) are phenotypic and genotypic covariance between traits x and y; σ^2^p_x_ and σ^2^p_y_ are phenotypic variances for traits x and y; σ^2^g_x_ and σ^2^g_y_ are genotypic variances for traits x and y, respectively.

### Path-coefficient analysis

Using path coefficient analysis, which was proposed by Dewey and Lu^27^ and developed by Wright^28^, the correlation coefficient was further divided into direct and indirect effects^28^. It is possible to tell whether a cause-and-effect relationship between two explanatory traits is real and independent of other traits by using path analysis. LY and GY were chosen for the path-coefficient analysis as the result (dependent) variables and the other traits as the causal (predictor) traits. The LY was also considered as an independent trait in path coefficient analysis for GY and vice versa. The direct and indirect effects of the independent traits on LY and GY were estimated by the simultaneous solution of the following equations which may be represented by the following general formula as applied by Dewey and Lu^27^.

3 $$rij=Pij+\sum rik*Pkj$$

where is the mutual association between the independent trait (i) and dependent trait (j) as measured by the genotypic and phenotypic correlation coefficients; is components of direct effects of the independent trait (i) on the dependent trait (j) as measured by the genotypic and phenotypic path-coefficients; and $\sum \mathrm{rik}*\mathrm{Pkj}$ is the summation of components of indirect effects of a given independent trait (i) on a given dependent trait (j) via all other independent traits (k). To determine values, square matrices of the correlation coefficients between independent traits in all possible pairs were inverted and then multiplied by the correlation coefficients between the independent and dependent traits.

Based on average data from the two years, path coefficient analysis was conducted using phenotypic and genotypic correlation matrices built up as X = Y − 1 * Z for LY and GY. The phenotypic and genotypic correlation coefficients of dependent traits (LY and GY) versus independent agro-morphological traits are represented in this matrix by the vector ′X. Vector Z is the direct effect of the route coefficients, and vector Y′ is the inversed value of the phenotypic and genotypic correlations for all conceivable combinations among the examined traits. The matrix inverse (MINVERSE) function of Microsoft Excel 2010 was used) compute the inverse of matrix Y′ after inverting the reciprocals and autocorrelations of the correlation coefficient matrix. Using the matrix multiplication (MMULT) function of Microsoft Excel 2010, the direct influence of path coefficients was estimated as the product of vector ′X and each row of vector Y′ inverse. As recommended, the value of the direct effect path coefficient was multiplied by the correlation coefficient in the matrix to assess the indirect effect of the path coefficient^27^. The direct effect product in the path analysis and the dependent trait coefficient in the correlation analysis via all predictor traits are added to create the coefficient of determination (R^2^). The contribution of the remaining unknown factor was measured as the residual factor (RF), which was calculated with the following formula;

4 $$\mathrm{RF}= \sqrt{1-{\mathrm{R}}^{2 }} ;\mathrm{ where }{\mathrm{R}}^{2 }=(\sum \mathrm{PIJ}*\mathrm{rij})$$

The value of RF reveals how effectively the causative factors explain the dependent factor's variability^29^. That is, if the RF value is low (for example, close to zero), the variability in the predictor characteristics fully accounts for the variance in the dependent trait, but a greater RF value suggests that other factors that have not been taken into account must be included in the study.

## Results

### Phenotypic and genotypic correlation of yield-related traits

Out of 91 relations, 71 yield-related trait relationships were significant at the phenotypic level, according to the linear pairwise correlations analysis (Table 3). The results revealed a very strong positive correlation between the LW and the LL (r_p_ = 0.90), LA (r_p_ = 0.77), DF (r_p_ = 0.61), PHF (r_p_ = 0.48), and PHM (r_p_ = 0.45). The correlation coefficients of TILL (r_p_ = 0.86), TISL (r_p_ = 0.80), and GSFR (r_p_ = 0.30) were also observed to be highly significant and positive with AIL. On the contrary, it revealed a very highly significant negative phenotypic correlation with PHM (r_p_ = −0.56), BN (r_p_ = −0.50), LN (r_p_ = −0.35), PHF (r_p_ = −0.54), DM (r_p_ = −0.42), and DF (r_p_ = −0.64). BN was significantly and positively correlated with PHM (r_p_ = 0.74), LN (r_p_ = 0.39), PHF (r_p_ = 0.66), DM (r_p_ = 0.70), DF (r_p_ = 0.58), and DE (r_p_ = 0.58), but very negatively correlated with TILL (r_p_ = −0.48) and GSFR (r_p_ = −0.36), as well as highly and negatively correlated with TISL (r_p_ = −0.18). The results also demonstrated a significant negative relationship between LL (rp = −0.20 ), and DE (rp = −0.21), with the AIL. The LL had a very high positive correlation with LW (r_p_ = 0.90), PHM (r_p_ = 0.50), PHF (r_p_ = 0.56), LA (r_p_ = 0.76), DF (r_p_ = 0.62), and GSFR (r_p_ = 0.29), but a high negative correlation with DE (r_p_ = −0.19). The AIL (r_p_ = 0.86), TISL (r_p_ = 0.78), and GSFR (r_p_ = 0.44) were shown to have a very highly significant positive connection with the TILL. With PHM (r_p_ = 0.78), BN (r_p_ = 0.66), LL (r_p_ = 0.56), DM (r_p_ = 0.60), LA (r_p_ = 0.32), DF (r_p_ = 0.82), and DE (r_p_ = 0.32), it was shown that the PHF had a very highly significant positive association with each of these variables. Strong positive associations with DM were seen for PHM (r_p_ = 0.52), BN (r_p_ = 0.70), LN (r_p_ = 0.32), PHF (r_p_ = 0.60), DF (r_p_ = 0.60), and DE (r_p_ = 0.60). The LA was identified to have a very highly significant negative association with DE (r_p_ = −0.34), but a very highly significant positive connection with LW (r_p_ = 0.77), LL (r_p_ = 0.76), and DF (r_p_ = 0.47). The DE was observed to have a very highly significant negative association with LA (r_p_ = −0.34) but a very highly significant positive relationship with PHM (r_p_ = 0.42), BN (r_p_ = 0.579), PHF (r_p_ = 0.32), and DM (rp = 0.36). DF was significantly positively correlated with BN (r_p_ = 0.58), LL (r_p_ = 0.62), PHF (r_p_ = 0.82), LA (r_p_ = 0.47), and DM (r_p_ = 0.82). AIL (r_p_ = 0.30), LL (r_p_ = 0.29), and TILL (r_p_ = 0.44) were found to have a very highly significant positive correlation with the GSFR, whereas BN (r_p_ = 0.36), LN (r_p_ = −0.31), and DM (r_p_ = 0.48 were found to have a very highly significant negative correlation with the GSFR.

**Table 3:**
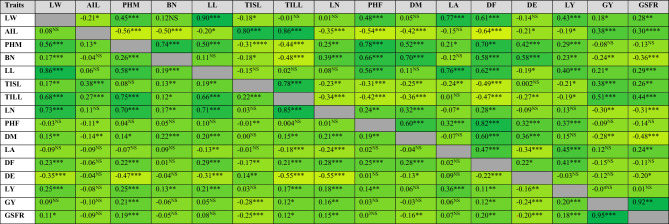
Phenotypic (above diagonal) and genotypic (below diagonal) correlations of 16 agro-morphological traits in 120 amaranth genotypes grown at Hawassa University agricultural research site in 2020 and 2021 cropping seasons.

Axillary inflorescence length (cm) = AIL, leaf yield (t/ha) = LY, grain yield (t/ha) = GY, days to maturation = DM, leaf length (cm) = LL, leaf width (cm) = LW, branch number (number) = BN, leaf number (number). LN, plant height at flowering stage (cm) = PHF, plant height at maturity (cm) = PHM, terminal inflorescence laterals length (cm) = TILL, terminal inflorescence stalk length (cm) = TISL, grain sink filling rate (kg ha^−1^ day^−1^) = GSFR. The green and yellow colors indicate the strength of correlation; green is positively correlated; green to light green indicates strong to weak positive correlation; yellow is negatively correlated, and yellow to light yellow indicates strong to weak negative correlation. Below the diagonal is the correlation coefficient; while above the diagonal is the significance level. ns = non- significant (p ≤ 0.05), *significant (p ≤ 0.05),**highly significant (p ≤ 0.05), ***very highly significant (p < 0.0001).

The analysis revealed that among the variables that contribute to yield, 60 associations out of a total of 91 relationships were significant at the genotypic level (Table 3). The findings showed that PHM (r_g_ = 0.56), BN (r_g_ = 0.17), LL (r_g_ = 0.86), TILL (r_g_ = 0.68), LN (r_g_ = 0.73), and DF (r_g_ = 0.23) all exhibited a very highly significant positive correlation with the LW. The opposite was true, however, as it showed a very strong negative genotypic relationship (r_g_ = −0.35) with DE. While the AIL displayed a highly significant and negative link with DM (r_g_ = −0.14), it had a very highly significant and positive correlation with TISL (r_g_ = 0.38) and TILL (r_g_ = 0.27) instead. The PHM showed very significant positive relationships with the following variables: BN (r_g_ = 0.26), LL (r_g_ = 0.58), TILL (r_g_ = 0.75), LN (r_g_ = 0.70), DF (r_g_ = 0.22), and GSFR (r_g_ = 0.19); however, the PHM also showed very high negative associations with DE (r_g_ = -0.47). With PHM (r_g_ = 0.26), LL (r_g_ = 0.19), and DM (r_g_ = 0.22), as well as with LN (r_g_ = 0.17), and TISL (r_g_ = 0.13), the BN showed a very strong positive association. The TISL had a very highly significant negative correlation with GSFR (r_g_ = −0.25) but a very highly significant positive correlation with AIL (r_g_ = 0.38) and TILL (r_g_ = 0.22). Concerning LW (r_g_ = 0.73), PHM (r_g_ = 0.70), LL (r_g_ = 0.71), DM (r_g_ = 0.21), and DF (r_g_ = 0.28), the LN exhibited very highly significant positive relationships. Conversely, it showed a very strong negative genotypic relationship with DE (r_g_ = -0.55) and LA (r_g_ = -0.24). The DM noted that BN (r_g_ = 0.22), LL (r_g_ = 0.20), LN (r_g_ = 0.21), and DF (r_g_ = 0.28) all had very highly significant positive correlations. The DF was considerably positively connected with LW (r_g_ = 0.23), PHM (r_g_ = 0.22), LL (r_g_ = 0.29), TILL (r_g_ = 0.21), LN (r_g_ = 0.28), PHF (r_g_ = 0.25), and DM (r_g_ = 0.28), but strongly negatively correlated with DE (r_g_ = −0.22). With LW (r_g_ = −0.35), PHM (r_g_ = −0.47), LL (r_g_ = −0.31), TILL (r_g_ = −0.55), LN (r_g_ = −0.55), DF (r_g_ = −0.22), and GSFR (r_g_ = −0.20), the DE displayed extremely strong negative associations. While TISL (r_g_ = −0.25) showed a very strong and negative association, the GSFR showed extremely substantial and positive correlations with PHM (r_g_ = 0.19).

### Yields' phenotypic and genotypic associations with other traits

In this study, LY and its yield-contributing characteristics were correlated in 15 ways, with 10 phenotypic associations and 12 genotypic relationships being significant (Table 3). The results showed that LY had positive phenotypic associations with LW (r_p_ = 0.43), PHM (r_p_ = 0.29), LL (r_p_ = 0.40), PHF (r_p_ = 0.37), LA (r_p_ = 0.45), DF (r_p_ = 0.41), and BN (r_p_ = 0.23), all of which were highly significant. AIL (r_p_ = −0.19), TISL (r_p_ = −0.21), and TILL (r_p_ = −0.19) all demonstrated a strong negative phenotypic connection with it as well. Additionally, LY had extremely strong positive genotypic correlations with PHM (r_g_ = 0.25), LL (r_g_ = 0.21), LN (r_g_ = 0.18), TILL (r_g_ = 0.17), LA (r_g_ = 0.36), GY (r_g_ = 0.20), and GSFR (r_g_ = 0.18). Additionally, it showed a strong positive genotypic correlation with the BN, PHF, and DF (r_g_ = 0.13, 0.14, and 0.11, respectively.

In the current study, GY and its yield-contributing characteristics had 17 significant correlations, of which 9 genotypic associations and 8 phenotypic relations were significant (Table 3). The findings revealed a very strong positive phenotypic correlation between GY and GSFR (r_p_ = 0.92), AIL (r_p_ = 0.38), TISL (r_p_ = 0.38), and TILL (r_p_ = 0.51). Additionally, it showed a substantial positive phenotypic connection with both LW and LL (r_p_ = 0.18 and 0.21, respectively). Additionally, it had a very strong negative phenotypic correlation (r_p_ = −0.24), (r_p_ = −0.30), and (r_p_ = −0.28) with BN, LN, and DM respectively. GSFR (r_g_ = 0.95), TISL (r_g_ = 0.28), PHM (r_g_ = 0.21), and LY (r_g_ = 0.21) likewise displayed a very highly significant positive genotypic correlation with the GY. Furthermore, it showed a highly significant positive genotypic association with LN (r_g_ = 0.16), a substantial positive genotypic association with DF (r_g_ = 0.12), and a significant positive genotypic association with TILL (r_g_ = 0.12).

### Phenotypic and genotypic path coefficients on leaf yield

Table 4 lists the estimations of the phenotypic effects, both direct and indirect, on LY in Amaranth genotypes. At the phenotypic level, the results showed that LA (0.3849; 47.93%) had the highest positive direct effect on LY, followed by AIL (0.4411; 34.283%), BN (0.3248; 30.17%), LW (0.1465; 17.894%), PHF (0.1420; 12.120%), and LN (0.0417; 7.135%). DM, DF, and GY were additional characteristics that had strong direct effects on LY. TILL (−0.3009; 26.735%), TISL (−0.1246; 12.059%), and PHM (−0.0976; 8.541%) all had detrimental direct effects on LY. Similarly, LL and DE directly have negative effects on LY. Except for AIL, BN, TISL, LN, DM, and DE, the examined characteristics had positive indirect effects on LY via LA. Likewise, GY had positive indirect effects on LY via LW, AIL, PHM, DM, LA, and DE. LW (0.2968; 36.248%), LL (0.2940; 35.758%), DF (0.1826; 14.206%), PHF (0.1230; 10.499%), PHM (0.0796; 6.960%), GY (0.0471; 7.717%), and TILL (0.0027; 0.243%) via LA all had the strongest positive indirect effects on LY. Through LA (0.2968; 36.248%), PHF (0.0681; 8.316%), BN (0.0397; 4.851%), DF (0.0554; 4.118%), TISL (0.0227; 2.777%), DE (0.0080; 0.976%), TILL (0.0044; 0.533%), GY (0.0023; 0.276%), and LN (0.0006; 0.072%), LW had positive indirect effects on LY. Likewise, the positive indirect effects of PHM were observed on LY via BN (0.2396; 20.959%), TILL (0.1317; 11.519%), PHF (0.1110; 9.713%), LW (0.0657; 5.746%), DF (0.0390; 3.415%), TISL (0.0381; 3.329%), and LN (0.0103; 0.903%).

**Table 4:** Estimated phenotypic direct (horizontal) and indirect (off-diagonal) effects of 14 traits on leaf yield of 120 amaranth genotypes in Hawassa University agricultural research site in 2020 and 2021 cropping seasons.

	Traits	LW	AIL	PHM	BN	LL	TISL	TILL	LN	PHF	DM	LA	DF	DE	GY
Path coefficients	Direct effect	0.1465	0.4411	−0.0976	0.3248	−0.0624	−0.1246	−0.3009	0.0417	0.1420	0.0562	0.3849	0.0554	−0.0569	0.0125
	Indirect effect via														
	LW		−0.0932	−0.0438	0.0397	−0.0562	0.0227	0.0044	0.0006	0.0681	−0.0029	0.2968	0.0337	0.0080	0.0023
	AIL	−0.0309		0.0544	−0.1639	0.0124	−0.1002	−0.2601	−0.0145	−0.0762	0.0237	−0.0572	−0.0356	0.0117	0.0048
	PHM	0.0657	−0.2456		0.2396	−0.0310	0.0381	0.1317	0.0103	0.1110	−0.0292	0.0796	0.0390	−0.0237	−0.0010
	BN	0.0179	−0.2226	−0.0720		−0.0072	0.0227	0.1444	0.0162	0.0942	−0.0391	−0.0474	0.0321	−0.0329	−0.0030
	LL	0.1319	−0.0875	−0.0485	0.0373		0.0183	−0.0063	0.0033	0.0790	−0.0059	0.2940	0.0344	0.0107	0.0027
	TISL	−0.0267	0.3549	0.0298	−0.0591	0.0092		−0.2350	−0.0094	−0.0446	0.0139	−0.0936	−0.0273	0.0001	0.0048
	TILL	−0.0021	0.3812	0.0427	−0.1559	−0.0013	−0.0973		−0.0141	−0.0592	0.0202	0.0027	−0.0261	0.0154	0.0064
	LN	0.0021	−0.1533	−0.0242	0.1258	−0.0049	0.0282	0.1018		0.0347	−0.0181	−0.0254	0.0155	0.0049	−0.0037
	PHF	0.0703	−0.2366	−0.0764	0.2155	−0.0347	0.0391	0.1254	0.0102		−0.0338	0.1230	0.0454	−0.0180	−0.0012
	DM	0.0075	−0.1856	−0.0507	0.2257	−0.0066	0.0308	0.1080	0.0134	0.0853		−0.0282	0.0332	−0.0204	−0.0035
	LA	0.1130	−0.0655	−0.0202	−0.0400	−0.0477	0.0303	−0.0021	−0.0028	0.0454	0.0041		0.0263	0.0192	0.0015
	DF	0.0891	−0.2836	−0.0688	0.1879	−0.0388	0.0613	0.1416	0.0117	0.1164	−0.0337	0.1826		−0.0128	−0.0019
	DE	−0.0206	−0.0905	−0.0407	0.1878	0.0118	0.0003	0.0813	−0.0036	0.0450	−0.0202	−0.1302	0.0125		−0.0015
	GY	0.0265	0.1678	0.0075	−0.0775	−0.0133	−0.0476	−0.1540	−0.0123	−0.0133	0.0158	0.0471	−0.0086	0.0069	
Percent path coefficients	Direct effect	17.894	34.283	8.541	30.171	7.594	12.059	26.735	7.135	12.120	6.575	47.930	4.311	8.097	2.046
	Indirect effect via														
	LW		11.377	5.346	4.851	6.862	2.777	0.533	0.072	8.316	0.354	36.248	4.118	0.976	0.276
	AIL	2.405		4.227	12.737	0.963	7.791	20.215	1.126	5.921	1.839	4.446	2.770	0.908	0.369
	PHM	5.746	21.487		20.959	2.714	3.329	11.519	0.903	9.713	2.554	6.960	3.415	2.076	0.084
	BN	1.665	20.677	6.693		0.667	2.107	13.419	1.500	8.755	3.631	4.402	2.979	3.058	0.277
	LL	16.035	10.642	5.899	4.538		2.223	0.772	0.398	9.605	0.722	35.758	4.184	1.306	0.323
	TISL	2.588	34.352	2.887	5.721	0.887		22.748	0.914	4.319	1.348	9.061	2.640	0.012	0.462
	TILL	0.189	33.870	3.796	13.849	0.117	8.645		1.253	5.259	1.793	0.243	2.318	1.366	0.568
	LN	0.356	26.243	4.140	21.535	0.840	4.832	17.425		5.936	3.090	4.348	2.657	0.831	0.633
	PHF	5.996	20.193	6.516	18.394	2.964	3.341	10.705	0.869		2.884	10.499	3.878	1.540	0.100
	DM	0.882	21.701	5.929	26.389	0.771	3.607	12.623	1.565	9.978		3.299	3.882	2.389	0.411
	LA	14.069	8.162	2.513	4.978	5.940	3.773	0.266	0.343	5.652	0.513		3.275	2.397	0.190
	DF	6.934	22.057	5.349	14.613	3.015	4.769	11.017	0.908	9.054	2.620	14.206		0.996	0.150
	DE	2.929	12.878	5.794	26.727	1.677	0.039	11.565	0.506	6.405	2.873	18.522	1.774		0.214
	GY	4.334	27.482	1.235	12.686	2.171	7.793	25.215	2.020	2.182	2.592	7.717	1.405	1.122	

R^2^ = 0.98341, residual factor = 0.12880.

*AIL* axillary inflorescence length, *GY* grain yield, *LL* leaf length, *LW* leaf width, *BN* branch number , *LN* leaf number, *PHF* plant height at flowering, *PHM* plant height at maturity , *TILL* terminal inflorescence laterals length, *TISL* terminal inflorescence stalk length , *LA* leaf area , *DF* days to flowering, *DE* days to emergence.

Table 5 shows the magnitudes of the direct and indirect genotypic effects of various traits on LY in Amaranth genotypes. LW (0.2225; 38.256%), PHF (0.1378; 73.869%), PHM (0.1277; 23.738%), GY (0.1264; 45.404%), LN (0.0969; 15.269%), TISL (0.0827; 11.643), and BN (0.0168; 8.513%) showed the next largest direct effects on LY after LA (0.3766; 78.608%). TILL (−0.0837; 13.441%), LL (−0.0672; 11.643%), AIL (−0.0760; 30.517%), and DM (−0.0166; 8.928%) on the other hand, had negative direct effects on LY. DE similarly showed adverse direct effects on LY. Despite having the largest positive direct effect on LY, LN, TISL, TILL, LL, LW, AIL, PHM, and DM had negative indirect effects through LA. BN (0.0351; 17.784%) had strong positive indirect effects on LY via LA, followed by DE (0.0352; 8.591%), GY (0.0218; 7.836%), DF (0.0078; 3.023%), and PHF (0.0066; 3.554%). Similar indirect effects via GY were seen for the following variables: DF (0.0157; 6.104%), PHM (0.0261; 4.858%), LN (0.0202; 3.184%), TILL (0.0110; 2.408%), PHF (0.0041; 2.215%), LW (0.0118; 1.527%), LA (0.0073; 2.408%), and LL (0.0069; 1.198%). But LN (−0.0898; 14.149%) had the most detrimental indirect effect on LY through LA.

**Table 5:** Estimated genotypic direct (horizontal) and indirect (off-diagonal) effects of 14 traits on leaf yield of 120 amaranth genotypes in Hawassa University agricultural research site in 2020 and 2021 cropping seasons.

	Traits	LW	AIL	PHM	BN	LL	TISL	TILL	LN	PHF	DM	LA	DF	DE	GY
Path coefficients	Direct effect	0.2225	−0.0760	0.1277	0.0168	−0.0672	0.0827	−0.0837	0.0969	0.1378	−0.0166	0.3766	−0.0171	−0.0626	0.1264
	Indirect effect via														
	LW		−0.0064	0.0720	0.0029	−0.0577	0.0138	−0.0571	0.0703	−0.0046	−0.0025	−0.0345	−0.0039	0.0217	0.0118
	AIL	0.0186		0.0171	−0.0006	−0.0039	0.0317	−0.0226	0.0110	−0.0148	0.0024	−0.0338	0.0011	0.0027	−0.0125
	PHM	0.1255	−0.0102		0.0043	−0.0390	0.0068	−0.0624	0.0678	0.0055	−0.0023	−0.0271	−0.0037	0.0295	0.0261
	BN	0.0386	0.0028	0.0329		−0.0131	0.0105	−0.0098	0.0168	0.0071	−0.0037	0.0351	−0.0002	0.0026	−0.0072
	LL	0.1910	−0.0044	0.0742	0.0033		0.0158	−0.0557	0.0684	0.0136	−0.0033	−0.0489	−0.0050	0.0195	0.0069
	TISL	0.0370	−0.0291	0.0105	0.0021	−0.0129		−0.0181	0.0034	−0.0011	0.0000	−0.0043	0.0029	−0.0089	−0.0355
	TILL	0.1518	−0.0205	0.0952	0.0020	−0.0447	0.0179		0.0825	−0.0006	−0.0025	−0.0686	−0.0037	0.0343	0.0150
	LN	0.1615	−0.0086	0.0893	0.0029	−0.0474	0.0029	−0.0713		0.0009	−0.0035	−0.0898	−0.0048	0.0347	0.0202
	PHF	−0.0074	0.0082	0.0051	0.0009	−0.0066	−0.0007	0.0004	0.0006		−0.0031	0.0066	−0.0043	−0.0007	0.0041
	DM	0.0340	0.0109	0.0177	0.0037	−0.0133	0.0000	−0.0125	0.0204	0.0261		−0.0144	−0.0047	0.0078	−0.0032
	LA	−0.0204	0.0068	−0.0092	0.0016	0.0087	−0.0009	0.0152	−0.0231	0.0024	0.0006		−0.0004	−0.0058	0.0073
	DF	0.0513	0.0048	0.0276	0.0002	−0.0196	−0.0139	−0.0179	0.0275	0.0349	−0.0046	0.0078		0.0138	0.0157
	DE	−0.0773	0.0033	−0.0603	−0.0007	0.0210	0.0117	0.0459	−0.0537	0.0014	0.0021	0.0352	0.0038		−0.0307
	GY	0.0207	0.0075	0.0264	−0.0010	−0.0037	−0.0232	−0.0099	0.0155	0.0045	0.0004	0.0218	−0.0021	0.0152	
Percent path coefficients	Direct effect	38.256	30.517	23.738	8.513	11.643	11.643	13.441	15.269	73.869	8.928	78.608	6.651	15.279	45.404
	Indirect effect via														
	LW		1.093	12.379	0.500	9.917	2.365	9.820	12.087	0.791	0.434	5.925	0.677	3.735	2.020
	AIL	7.483		6.885	0.253	1.558	12.750	9.082	4.426	5.956	0.956	13.573	0.435	1.096	5.031
	PHM	23.327	1.895		0.804	7.256	1.264	11.604	12.595	1.019	0.427	5.034	0.687	5.492	4.858
	BN	19.569	1.445	16.695		6.631	5.324	4.985	8.502	3.591	1.868	17.784	0.120	1.339	3.633
	LL	33.103	0.759	12.853	0.566		2.744	9.645	11.847	2.351	0.566	8.478	0.862	3.384	1.198
	TISL	14.895	11.728	4.225	0.857	5.176		7.283	1.361	0.460	0.003	1.713	1.157	3.574	14.271
	TILL	24.370	3.292	15.282	0.316	7.171	2.870		13.247	0.104	0.396	11.010	0.587	5.505	2.408
	LN	25.443	1.361	14.069	0.458	7.470	0.455	11.236		0.137	0.549	14.149	0.762	5.460	3.184
	PHF	3.985	4.382	2.725	0.463	3.548	0.368	0.211	0.327		1.683	3.554	2.320	0.349	2.215
	DM	18.318	5.892	9.568	2.015	7.147	0.022	6.734	11.003	14.090		7.782	2.544	4.219	1.738
	LA	4.249	1.422	1.916	0.326	1.822	0.195	3.182	4.821	0.506	0.132		0.073	1.220	1.527
	DF	20.001	1.876	10.761	0.091	7.628	5.428	6.987	10.699	13.601	1.782	3.023		5.370	6.104
	DE	18.867	0.809	14.724	0.173	5.121	2.867	11.207	13.104	0.350	0.505	8.591	0.918		7.486
	GY	7.436	2.705	9.489	0.342	1.321	8.342	3.571	5.568	1.619	0.152	7.836	0.761	5.454	

R^2^ = 0.9851; residual factor = 0.1221.

*AIL* axillary inflorescence length, *GY* grain yield, *LL* leaf length, *LW* leaf width, *BN* branch number, *LN* leaf number, *DM* days to maturation, *PHF* plant height at flowering, *PHM* plant height at maturity, *TILL* terminal inflorescence laterals length, *TISL* terminal inflorescence stalk length, *LA * leaf area, *DF* days to flowering, *DE* days to emergence.

### Phenotypic and genotypic path coefficients on grain yield

Table 6 lists the assessed traits' phenotypic direct and indirect effects on GY. According to the findings, the variables that had the largest positive direct effects on GY were GSFR (0.9379; 79.845), BN (0.1825; 0.1825%), PHF (0.141; 0.1401%), TILL (0.1620; 17.694%), PHM (0.0678; 9.398%), AIL (0.661; 7.839%), and LA (0.0193; 0.0016%). The negative direct effects on GY were also exerted by DF (−0.1182; 16.302%), LW (−0.0789; 13.054%), LY (−0.0306; 10.146%), DE (−0.0452; 8.731%), LL (−0.0349; 5.591%), TISL (−0.0325; 5.053%), and LN (−0.0195; 3.464%). Despite the high positive direct effects of GSFR on GY, its indirect effects via LW, PHM, BN, LL, TISL, PHF, and, LY were negative. Along with the strongest positive direct effect on GY, the GSFR also had positive indirect effects via AIL, TILL, LN, LA, DF, and DE. BN positively affected GY both directly and indirectly through PHM, TISL, and PHF. The biggest positive indirect effects via BN were obtained by PHM (0.1347; 18.653%), PHF (0.11; 16.281%), DF (0.1056; 14.567%), LY (0.41; 13.822%), LN (0.07; 12.588%), LW (0.02; 3.694%), and LL (0.210; 3.358%). DE came in second (0.1056; 20.406%). TILL (0.4130; 45.127%), LA (0.2258; 45.042%), LL (0.2679; 42.887%), LW (0.2590; 42.855%), and TISL (0.2446; 37.999%) all contributed high and positive indirect effects via GSFR. However, BN had the largest negative indirect effects through GSFR, followed by LN, DE, PHF, PHM, and DF.

**Table 6:** Estimated phenotypic direct (horizontal) and indirect (off-diagonal) effects of 14 traits on grain yield of 120 amaranth genotypes in Hawassa University agricultural research site in 2020 and 2021 cropping seasons.

	Traits	LW	AIL	PHM	BN	LL	TISL	TILL	LN	PHF	LA	DF	DE	LY	GSFR
Path coefficients	Direct effect	−0.0789	0.0661	0.0678	0.1825	−0.0349	−0.0325	0.1620	−0.0195	0.1401	0.0016	−0.1182	−0.0452	−0.0306	0.9379
	Indirect effect via														
	LW		−0.0140	0.0304	0.0223	−0.0314	0.0059	−0.0023	−0.0003	0.0672	0.0013	−0.0719	0.0063	−0.0130	0.2590
	AIL	0.0167		−0.0378	−0.0921	0.0069	−0.0262	0.1400	0.0068	−0.0752	−0.0002	0.0760	0.0093	0.0058	0.2844
	PHM	−0.0354	−0.0368		0.1347	−0.0173	0.0099	−0.0709	−0.0048	0.1096	0.0003	−0.0832	−0.0188	−0.0088	−0.1235
	BN	−0.0096	−0.0334	0.0501		−0.0040	0.0059	−0.0777	−0.0075	0.0930	−0.0002	−0.0684	−0.0261	−0.0070	−0.3360
	LL	−0.0710	−0.0131	0.0337	0.0210		0.0048	0.0034	−0.0015	0.0779	0.0012	−0.0733	0.0085	−0.0122	0.2679
	TISL	0.0144	0.0532	−0.0207	−0.0332	0.0051		0.1265	0.0044	−0.0440	−0.0004	0.0581	0.0001	0.0063	0.2446
	TILL	0.0011	0.0571	−0.0297	−0.0876	−0.0007	−0.0254		0.0066	−0.0584	0.0000	0.0556	0.0122	0.0058	0.4130
	LN	−0.0011	−0.0230	0.0168	0.0707	−0.0027	0.0074	−0.0548		0.0342	−0.0001	−0.0331	0.0039	−0.0038	−0.2907
	PHF	−0.0378	−0.0355	0.0530	0.1211	−0.0194	0.0102	−0.0675	−0.0048		0.0005	−0.0969	−0.0143	−0.0113	−0.1314
	LA	−0.0608	−0.0098	0.0140	−0.0225	−0.0267	0.0079	0.0011	0.0013	0.0448		−0.0561	0.0153	−0.0136	0.2258
	DF	−0.0480	−0.0425	0.0478	0.1056	−0.0217	0.0160	−0.0762	−0.0054	0.1149	0.0008		−0.0102	−0.0124	−0.1052
	DE	0.0111	−0.0136	0.0283	0.1056	0.0066	0.0001	−0.0437	0.0017	0.0444	−0.0006	−0.0266		0.0008	−0.1893
	LY	−0.0337	−0.0126	0.0195	0.0416	−0.0140	0.0068	−0.0305	−0.0024	0.0519	0.0007	−0.0480	0.0012		0.0078
	GSFR	−0.0218	0.0200	−0.0089	−0.0654	−0.0100	−0.0085	0.0713	0.0060	−0.0196	0.0004	0.0133	0.0091	−0.0003	
Percent path coefficients	Direct effect	13.054	7.839	9.398	20.246	5.591	5.053	17.694	3.464	18.839	0.324	16.302	8.731	10.146	79.845
	Indirect effect via														
	LW		2.311	5.034	3.694	5.201	0.982	0.389	0.046	11.121	0.207	11.897	1.050	2.159	42.855
	AIL	1.975		4.480	10.921	0.822	3.103	16.598	0.802	8.914	0.029	9.008	1.099	0.691	33.720
	PHM	4.898	5.100		18.653	2.403	1.376	9.817	0.668	15.179	0.047	11.528	2.609	1.214	17.110
	BN	1.070	3.700	5.553		0.445	0.657	8.623	0.836	10.316	0.022	7.583	2.898	0.773	37.277
	LL	11.365	2.100	5.396	3.358		0.764	0.547	0.245	12.480	0.199	11.743	1.365	1.961	42.887
	TISL	2.236	8.263	3.220	5.160	0.796		19.650	0.685	6.841	0.061	9.033	0.015	0.986	37.999
	TILL	0.125	6.242	3.244	9.571	0.081	2.775		0.719	6.382	0.001	6.077	1.333	0.629	45.127
	LN	0.199	4.091	2.992	12.588	0.489	1.312	9.754		6.093	0.019	5.890	0.686	0.681	51.744
	PHF	5.084	4.767	7.131	16.281	2.611	1.374	9.074	0.639		0.070	13.020	1.925	1.521	17.663
	LA	12.131	1.959	2.797	4.481	5.320	1.578	0.229	0.256	8.933		11.182	3.046	2.721	45.042
	DF	6.621	5.863	6.592	14.567	2.990	2.208	10.517	0.752	15.849	0.106		1.401	1.714	14.518
	DE	2.142	2.622	5.469	20.406	1.274	0.014	8.456	0.321	8.588	0.106	5.137		0.151	36.585
	LY	11.177	4.185	6.460	13.822	4.647	2.243	10.125	0.808	17.229	0.241	15.951	0.382		2.584
	GSFR	1.854	1.706	−0.761	5.567	0.849	0.722	6.072	0.513	1.671	0.033	1.129	0.776	0.022	

R^2^ = 0.9899; residual factor = 0.1005.

*AIL* axillary inflorescence length, *LY* leaf yield, *GY* grain yield, *LL* leaf length, *LW* leaf width, *BN* branch number, *LN* leaf number, *PHF* plant height at flowering, *PHM* plant height at maturity, *TILL* terminal inflorescence laterals length, *TISL* terminal inflorescence stalk length, *GSFR* grain sink filling rate, *LA* leaf area, *DF* days to flowering, *DE* days to emergence.

Table 7 lists the examined traits' genotypic effects on GY, both directly and indirectly. GSFR (0.9297; 92.706%) had the most positive direct effect on GY, followed by PHF (0.0215; 23.943%), PHM (0.0533; 14.498%), and LN (0.0358; 10.385%). On the contrary, the highest negative direct effect on GY was exerted by DF (−0.0957; 27.051%), followed by BN (−0.0206; 18.309%), DE (−0.0531; 15.039%), TISL (−0.0420; 12.860%), TILL (−0.0354; 11.320%), LL (−0.0260; 9.961) and LA (−0.0112; 9.125%). GSFR had a highly positive direct effect on GY, whereas it had negative indirect effects via DF, TILL, LL, LW, and LA. PHM's positive direct effects on GY were accompanied by positive indirect effects through LW, AIL, BN, LL, TISL, TILL, and DF. The PHF had a positive direct effect on GY, and through BN, LL, LA, DF, and DE, this trait also had negative indirect effects on GY. For DF (0.0055; 1.542%), LY (0.0030; 1.139%), LL (0.0021; 0.814%), BN (0.0011; 0.983%), PHM (0.0009; 0.233%), GSFR (0.0718; 0.072%), LA (0.0004; 0.309%), DE (0.0002; 0.063%), and LN (0.0001; 0.039%), the highest positive indirect effects via PHF were observed. The highest positive indirect effects via GSFR were recorded for LY (0.1661; 62.387%), DF (0.1889; 53.371%), LA (0.0654; 53.246%), PHM (0.1736; 47.216%), LN (0.1408; 40.816%), LW (0.1047; 36.799%), TILL (0.1081; 34.620%), PHF (0.0310; 34.414%), and LL (0.0742; 28.449%). On the contrary, the highest negative indirect effects via GSFR were observed in TISL, AIL, BN, and DE.

**Table 7:** Estimated genotypic direct (horizontal) and indirect (off-diagonal) effects of 14 traits on grain yield of 120 amaranth genotypes in Hawassa University agricultural research site in 2020 and 2021 cropping seasons.

	Traits	LW	AIL	PHM	BN	LL	TISL	TILL	LN	PHF	LA	DF	DE	LY	GSFR
Path coefficients	Direct effect	−0.0155	−0.0056	0.0533	−0.0206	−0.0260	−0.0420	−0.0354	0.0358	0.0215	−0.0112	−0.0957	−0.0531	0.0344	0.9297
	Indirect effect via														
	LW		−0.0005	0.0301	−0.0036	−0.0223	−0.0070	−0.0241	0.0260	−0.0007	0.0010	−0.0221	0.0184	0.0085	0.1047
	AIL	−0.0013		0.0072	0.0008	−0.0015	−0.0161	−0.0095	0.0041	−0.0023	0.0010	0.0061	0.0023	−0.0027	−0.0813
	PHM	−0.0087	−0.0008		−0.0053	−0.0151	−0.0035	−0.0264	0.0250	0.0009	0.0008	−0.0207	0.0251	0.0086	0.1736
	BN	−0.0027	0.0002	0.0137		−0.0051	−0.0053	−0.0042	0.0062	0.0011	−0.0010	−0.0013	0.0022	0.0044	−0.0444
	LL	−0.0133	−0.0003	0.0310	−0.0040		−0.0080	−0.0235	0.0253	0.0021	0.0015	−0.0279	0.0166	0.0072	0.0742
	TISL	−0.0026	−0.0022	0.0044	−0.0026	−0.0050		−0.0076	0.0012	−0.0002	0.0001	0.0161	−0.0075	0.0010	−0.2338
	TILL	−0.0106	−0.0015	0.0397	−0.0024	−0.0173	−0.0091		0.0305	−0.0001	0.0020	−0.0205	0.0291	0.0060	0.1081
	LN	−0.0112	−0.0006	0.0373	−0.0036	−0.0183	−0.0015	−0.0301		0.0001	0.0027	−0.0271	0.0294	0.0063	0.1408
	PHF	0.0005	0.0006	0.0021	−0.0011	−0.0026	0.0003	0.0002	0.0002		−0.0002	−0.0243	−0.0006	0.0048	0.0310
	LA	0.0014	0.0005	−0.0038	−0.0019	0.0034	0.0005	0.0064	−0.0085	0.0004		−0.0020	−0.0050	0.0124	0.0654
	DF	−0.0036	0.0004	0.0115	−0.0003	−0.0076	0.0071	−0.0076	0.0101	0.0055	−0.0002		0.0117	0.0038	0.1889
	DE	0.0054	0.0002	−0.0252	0.0009	0.0081	−0.0060	0.0194	−0.0198	0.0002	−0.0010	0.0211		−0.0055	−0.1872
	LY	−0.0038	0.0004	0.0132	−0.0027	−0.0054	−0.0012	−0.0062	0.0066	0.0030	−0.0040	−0.0106	0.0085		0.1661
	GSFR	−0.0017	0.0005	0.0100	0.0010	−0.0021	0.0106	−0.0041	0.0054	0.0007	−0.0008	−0.0195	0.0107	0.0062	
Percent path coefficients	Direct effect	5.4420	3.9795	14.498	18.309	9.961	12.860	11.320	10.385	23.943	9.125	27.051	15.039	12.924	92.706
	Indirect effect via														
	LW		0.166	10.565	1.256	7.840	2.453	8.478	9.135	0.253	0.360	7.765	6.483	3.004	36.799
	AIL	0.913		5.041	0.545	1.057	11.347	6.728	2.870	1.634	0.708	4.279	1.632	1.928	57.337
	PHM	2.375	0.206		1.446	4.105	0.938	7.170	6.811	0.233	0.219	5.634	6.823	2.327	47.216
	BN	2.384	0.188	12.204		4.490	4.731	3.687	5.503	0.983	0.926	1.182	1.991	3.950	39.472
	LL	5.096	0.125	11.872	1.538		3.080	9.013	9.690	0.814	0.558	10.699	6.357	2.749	28.449
	TISL	0.789	0.664	1.342	0.802	1.524		2.341	0.383	0.055	0.039	4.941	2.310	0.304	71.648
	TILL	3.381	0.488	12.722	0.775	5.530	2.904		9.765	0.032	0.653	6.567	9.322	1.922	34.620
	LN	3.257	0.186	10.806	1.034	5.315	0.425	8.731		0.039	0.774	7.868	8.530	1.833	40.816
	PHF	0.575	0.675	2.358	1.178	2.845	0.387	0.185	0.251		0.219	26.970	0.614	5.387	34.414
	LA	1.154	0.413	3.122	1.563	2.749	0.386	5.245	6.956	0.309		1.607	4.042	10.083	53.246
	DF	1.009	0.101	3.257	0.081	2.139	1.997	2.139	2.867	1.542	0.065		3.306	1.074	53.371
	DE	1.522	0.070	7.126	0.246	2.296	1.686	5.487	5.615	0.063	0.296	5.972		1.567	53.015
	LY	1.443	0.169	4.973	1.000	2.032	0.454	2.315	2.470	1.139	1.513	3.973	3.207		62.387
	GSFR	0.174	0.049	0.992	0.098	0.207	1.053	0.410	0.541	0.072	0.078	1.940	1.067	0.613	

R^2^ = 0.987; residual factor = 0.1140.

*AIL* axillary inflorescence length, *LY* leaf yield, *GY* grain yield, *LL* leaf length, *LW* leaf width, *BN* branch number, *LN* leaf number, *PHF* plant height at flowering, *PHM* plant height at maturity, *TILL* terminal inflorescence laterals length, *TISL* terminal inflorescence stalk length, *GSFR* grain sink filling rate, *LA* leaf area, *DF* days to flowering, *DE* days to emergence.

## Discussion

Understanding the relationships between characters is crucial for any crop improvement effort since it indirectly influences selection success. To find the component characters that can be used in selection to boost yield, a correlation analysis is conducted to evaluate the underlying correlations between distinct characters. The correlation coefficient measures the degree to which two variables are positively or negatively associated. In the current trial, from the two sets of correlation coefficients, 61(50.83%) of the 120 possible pairs of trait comparisons across traits showed that the genotypic correlation coefficient was higher than the corresponding phenotypic correlation coefficient, which showed that the association was largely due to genetic factors among various traits, and that enhanced genetic inherent association^30–32^**,** this aids in determining the attributes that will be used in the breeding scheme. Moreover, genetic correlations between two characters arise because of linkage, pleiotropic or developmentally induced functional relationships^33^. As a result, it is more important and may be used to build a successful selection system. However, the remaining pairs had higher phenotypic correlation coefficients corresponding to genotypic correlation coefficients suggesting that environmental factors influenced the inherent associations among the different traits under study.

The phenotypic correlation analysis indicated that the LW was associated significantly and positively with LL which also correlated significantly and positively with PHM, PHF, LA, and DF. Other research findings also showed that the LL had a significant positive association with LW and PHM, and significant positive associations with LA were reported at the phenotypic level^34,35^. The LL had a very high positive correlation with LW, PHM, PHF, LA, DF, and GSFR. Similar to LW and LA, Jangde et al.^34^ found a substantial positive association between leaf length and both. In the current finding of PHM, BN, LL, DM, LA, DF, and DE, it was shown that the PHF had a very highly significant positive association with each of these variables. Singh^36^, discovered comparable results in the previous study on amaranth genotypes for plant height exhibited positive significant phenotypic correlations with days to 50% flowering and number of branches. The strong and positive link between leaf width and length also raises the prospect of enhancing both characteristics at the same time. A similar finding was reported by Olusanya^37^. BN was significantly and positively correlated with LN, PHM, and PHF, which indicates that selecting genotypes with taller plant heights would result in a greater number of branches, which is necessary for the production of leaf yields. Akter et al.^38^, came to the same results. DF was significantly and positively correlated with BN, PHF, and PHM. Similar findings were reported by Singh^36^. Days to maturity showed a positive and highly significant correlation with plant height. Similar results were reported by Shrivastav et al.^39^. The time of flowering are important adaptive trait in grain amaranth^15,40^. It was observed that leaf number and leaf area correlated negatively, suggesting that the more leaves amaranth produced, the smaller those leaves were. A negative correlation between the number of leaves and leaf area might act as a challenge in the improvement of leaf yield in amaranths. Furthermore, this pattern suggests that the length of the leaf and the area of the leaf decrease as the number of leave increase. Breeding strategies should focus on increasing the area of each leaf or on producing more leaves in each main branch. Similar results were reported by Gerrano et al.^41^.

Genotypic correlation between traits is important for identifying the most and least, important characteristics to be considered for the success of selection in breeding^42^. At the genotypic level, genes governing two separate traits are correlated positively at the coupling phase of linkage and negatively at the repulsive phase of linkage^43^. The relationship between leaf length and leaf width is very strong. Similarly, most of the leaf traits showed high positive correlations with one another, indicating that these traits are crucial for improving and selecting amaranth yields. The results of the trait correlations in this study imply that selection for these features in amaranth genotypes will be efficient and successful. Similar findings on garden peas were reported by Sharma and Sharma^44^. With the following variables BN, LL, TILL, LN, DF, and GSFR, the PHM demonstrated highly significant positive relationships. Similar results were reported by Shrivastav et al.^39^, and inflorescence length and days to 50% flowering were positively and extremely significantly correlated with plant height, according to Yadav et al.^45^. A positive and significant correlation with lateral inflorescence length, leaf length, leaf width, number of branches per plant, and days to maturity was obtained^46^. Similar results were found by Varalakshmi and Pratap Reddy^47^.

For the leaf yield, the phenotypic correlation studies carried out for this investigation revealed a strong and positive association between economically important parameters such as LA, LW, and LL. The results show that any enhancement of these characteristics will increase the potential LY of amaranth. Similar trends were identified by Kumar^48^, in their analysis. Therefore, consideration for these traits should be given in amaranth breeding programs. LY had significant genotypic and phenotypic relationships with LW, PHM, LL, PHF, LA, DF, and BN. The taller genotypes in the current study produced more leaves, which resulted in higher leaf yields; hence PHM as well as PHF did forecast the genotypes' high leaf-yielding potential due to their significant association. However, the fact that PHM and PHF had a negative association with GY suggested that taller genotypes had poor grain yields. Therefore, selecting a genotype for higher leaf yield by focusing on the taller genotypes and shorter genotypes would produce good grain yields. As a result, the breeding strategy gave higher priority to characters that increased LW, PHM, LL, PHF, LA, DF, and BN, increasing the chances of obtaining a genotype with a high leaf yield. Similar results were reported in amaranth genotypes by Varalakshmi and Pratap Reddy^47^, who reported that plant height, leaf length, and leaf width were positively and significantly correlated with the leaf yield. Moreover, choosing genotypes with taller plant heights will result in a better number of branches, which is required for the production of leafy vegetables, according to the statistically significant positive association between plant height and the number of branches. A similar finding was reported by Olaniyi^49^ Vegetable amaranths are said to benefit from late flowering and maturation since farmers would have more time to harvest the leaves^50^. A single gene controls early flowering in amaranth, and the dominant allele determines early flowering^51^. For grain production, early flowering and maturity would be preferable. The positive and significant correlations between yield and yield components in amaranth were also reported by^52,53^**.**

The results showed that at the phenotypic level, GY with GSFR, AIL, TISL, and TILL have extremely high positive phenotypic correlations. Furthermore, it demonstrated a strong positive phenotypic relationship with both LW and LL. The results suggest that these traits might be simultaneously enhanced to raise grain yield either individually or in combination. Showemimo et al.^54^, reported comparable results. As a result, amaranth should be chosen for future breeding programs to ensure the maximum exploitation of grain amaranth, as the photosynthetic capacity of a plant with longer leaves is predicted to be higher than that of a plant with shorter leaves^55^, and traits like a high leaf area, leaf length, and plant height enable optimal crop output when water is accessible as an essential agricultural input in sufficient quantities^56^. Grain yield exhibited a highly significant and positive phenotypic association with inflorescence length (TISL, TILL, and AIL). Nyasulu et al.^57^, Kumar^58^, and Yadav et al.^45^, obtained consistent findings in amaranth species. As a result, characteristics may be prioritized during selection to produce genotypes with higher grain yield.

The genotypic relationship between GSFR, PHM, and LY with the GY was also very substantial and positive. It also revealed a highly significant positive genotypic association with LN, a substantial positive genotypic association with DF, and a significant positive genotypic link with TILL. These findings demonstrated that selection for any one of these traits that contribute to grain yield will result in increases in the other traits, thereby finally boosting the grain yield. To select genotypes with higher grain production, primary selection for characteristics like GSFR, PHM, and LY may be prioritized. There were similar trends for the association of GY with other traits (PHM, TILL, and DF) that may be prioritized^45^. Grain yield and leaf area, length, and width are strongly correlated with one another. Similar to grain yield, all leaf traits showed high positive correlations with one another, indicating that these features are important for selecting amaranth leaf and grain productivity. The findings of the trait correlations in this study imply that selection for GSFR, PHM, and LY will be efficient and beneficial in improving grain production in grain amaranth genotypes, as also found in garden peas by Sharma and Sharma^44^. In addition, the positive correlation between grain yields and days of flowering revealed that early flowering would be a viable choice for a greater grain yield in this scenario. These results appear to be in line with a general pattern that early blooming is linked to higher grain yields across species^37,59^. These findings appear inconsistent with a general trend for late flowering to be associated with high grain yield across species^37^.

The relationship between any two features is merely depicted by their correlation coefficients, which do not reveal any potential underlying causes. The degree of the relationship between the yield and yield components can be determined by path coefficient analysis, which also helps to clarify the cause-and-effect interactions between different characters. Path coefficient analysis was performed to segregate the correlation coefficient into the direct and indirect effects of various features on yield. The yield-contributing features for LY and GY's varied phenotypic and genotypic correlation coefficients were further divided into direct and indirect effects. According to the findings, both phenotypic and genotypic levels of LY were positively affected directly by the LW, BN, LN, PHM, LA, and GY. This suggests that these traits can be used to develop a selection index that is both maximally reliable and effective at improving the leaf yield of amaranth genotypes. These findings confirmed the results of Aycicek and Yildirim^60^, for PHF in wheat and amaranths genotypes^48,61^, for the number of leaves in amaranths genotypes, Islam^62^ for leaf area, and Jangde et al.^34^ for leaf width . LL, TILL, and DE, on the other hand, were found to have the greatest negative direct effects on LY, both at the phenotypic and genotypic levels, showing that direct selection of these traits did not improve LY in amaranth genotypes. Following the present findings, the LL had a negative direct influence on LY in amaranth genotypes^35,48,62^, However, since PHM, LL, and TILL had such strong indirect effects, mostly through LA, while DM and DE exhibited such strong indirect effects, primarily through PHF, it was anticipated that LY in amaranth genotypes may also rise through an indirect selection of these traits. While TISL had positive direct effects on LY at the genotypic level, AIL had positive direct effects on LY solely at the phenotypic level. Additionally estimated was the residual effect, which establishes the unaccounted variability of the dependent variables (LY and GY). The characteristics that were included in the phenotypic and genotype path coefficient analyses explained 87.12% and 87.79% of the total variance in LY, respectively, according to the residual effects of 0.1288 and 0.1221. These results suggest that the independent variables taken into account in this study effectively represented the diversity of LY in amaranth genotypes. Other factors (at phenotypic level 12.88% and genotypic level 12.21%) that caused variations in LY, but were not taken into account in the current investigation, did exist.

A path analysis' findings revealed the importance of GSFR, PHM, and PHF traits for direct selection since they had considerable positive direct effects on GY both at the phenotypic and genotypic levels. This suggests that GY improvement in amaranth genotypes might be obtained through selection based on these characteristics. The result was congruity with^35,48^ for the plant height in amaranth genotypes. On the other hand, LW, TISL, LL, and DF had negative direct effects at both phenotypic and genotypic levels indicating that direct selection based on these traits could not improve the GY in amaranth genotypes. The results were also in agreement with the findings of Shrivastav et al.^39^, who reported that DF had a negative direct effect on GY in amaranth genotypes. The direct selection of LW, TISL, LL, and DF at both phenotypic and genotypic levels would decrease the GY; however, their indirect selection through mainly PHM may improve the GY in amaranth genotypes. PHM had beneficial indirect effects on LW, LL, TISL, and DF in addition to its beneficial direct effects on GY. For the indirect selection of high seed-yielding genotypes in amaranths, the DF may be a trustworthy indicator. Alake and Otusanya^63^, obtained consistent findings in amaranths genotypes. The direct effect of an AIL, BN, TILL, LA, and DE on GY was negative only at the genotypic level while LN and LY had a positive direct effect on GY only at the genotypic level. Other research findings also showed that LN had a positive direct effect at the genotypic level on GY in amaranth genotypes^64^. While LY had a negative direct effect on GY only at the phenotypic level, AIL, BN, TILL, and LA had positive direct effects on GY only at the phenotypic level. On grain yield in amaranth genotypes, the same observation was made for TILL, BN, LA, and AIL^65^. Furthermore, LW, AIL, BN, TISL, TILL, LN, DF, LY, and GSFR might be indirectly selected, mostly through PHM, to improve the GY at the genotypic level. According to the residual values of 0.1005 and 0.1140, the traits included in the phenotypic and genotypic path coefficient analyses, respectively, explained 89.95% and 88.6% of the overall variation in GY. This suggests that additional traits may also be influencing GY.

When combined with path coefficients, correlation coefficients offer more accurate data that may be successfully predicted in crop development initiatives^66^. The findings indicated that selection based on the phenotypic expression of LW, BN, LN, PHF, LA, and GY could be successful for LY improvement in amaranth genotypes these traits had positive direct effects on LY along with a significant positive association with LY at both phenotypic and genotypic levels. On the other hand, LL demonstrated negative direct effects on LY both at the phenotypic and genotypic levels, even though LL, DF, and GSFR had significant positive phenotypic and genotypic associations with LY. The occurrence of significant positive phenotypic and genotypic associations along with inverse direct effects between LY and these traits could be due to the high positive counterbalancing potential of the phenotypic and genotypic indirect effects exerted by these traits via mainly LN. The genotypic direct effect of DM on LY was negative despite the non-significant positive genotypic association between the two traits, indicating that the positive genotypic association between these traits was caused by the high and positive neutralizing genotypic indirect effect of this via chiefly by LA. Despite AIL's positive phenotypic direct effect on LY, its negative phenotypic correlation with LY might be because of AIL's significant negative phenotypic indirect effect, which largely acts through LA.

The GSFR had significant positive phenotypic and genotypic relationships with GY as well as positive direct impacts on GY, suggesting that selection for genotypes of amaranth based on the phenotypic expression of these traits may be successful in improving GY. Wei et al.^67^ obtained consistent findings in rice, while Knott and Gebeyehou^68^**,** reported equivalent results in bread wheat. Likewise, GSFR, PHF, and LY exhibited significant positive genotypic associations with GY as well as positive genotypic direct effects, revealing that genotypic selection based on the phenotypic expression of these traits would be effective for enhancing GY in amaranth genotypes. Although PHM and PHF had positive phenotypic and genotypic direct effects on GY, their correlations with GY were negative and insignificant on both a phenotypic and genotypic level. This might be because PHM and PHF's indirect actions, which primarily affect BN, LL, and DF, have significant and detrimental neutralizing potential. The phenotypic and genotypic direct effects of LW, LL, and TISL were negative but their associations with GY were positive at genotypic levels. This was due to the high and positive neutralizing potential of the genotypic indirect effects exerted by both traits via mainly PHM, LN, LA, and LY. The phenotypic (DE) and genotypic (DF and DE) direct effects on GY and their respective associations with GY were negative and insignificant indicating that the GY improvement based on the selection of these traits could not be effective in amaranth genotypes.

## Conclusions

Information from correlation may be augmented with the use of route analysis to select features that significantly increase the grain and leaf yield of amaranth genotypes and hence enhance selection efficiency in breeding. The studies of phenotypic and genotypic correlation and path coefficients revealed that LA was the variable that was most crucial for the indirect selection of LY since it exhibited a strong positive direct influence on LY as well as a substantial positive correlation with LY. Furthermore, it was suggested that LY in amaranth genotypes may also rise by an indirect selection of PHM, LL, and TILL, which exhibited such potent indirect influences, mostly through LA, and DM and DE, which showed such strong indirect effects, primarily through PHF. Therefore, this study pointed out that traits that showed significant positive indirect impacts via LA should also be taken into account as indirect selection criteria for LY improvement in amaranth genotypes. Similarly, the studies of correlation and path coefficients demonstrated strong direct impacts of GSFR on GY, and noteworthy positive correlations of both traits with GY were seen at the phenotypic and genotypic levels. Additionally, PHF strongly influenced GY at the genotypic level and had a substantial positive correlation with GY. At both the phenotypic and genotypic levels, the GSFR also had a significant indirect favorable impact on GY, mostly through DF and LY. This showed that selection mostly focused on DF, LY, and GSFR would ultimately enhance the GY in amaranth genotypes. However, because DE has detrimental direct impacts on LY and GY at the genetic level, breeding progress in these genotypes might be negatively impacted.

## Data Availability

We declare that the data used in this manuscript is available if anyone desires to access request the corresponding author.

## References

[1] Sarker U, Islam MS, Rabbani MG, Oba S (2015). Variability, heritability and genetic association in vegetable amaranth (*Amaranthus tricolor* L.). Span. J. Agric. Res..

[2] Breene W (1991). Food uses of grain amaranth. Cereal Foods World.

[3] Mujica A, Jacobsen S (2003). The genetic resources of Andean grain amaranths (*Amaranthus caudatus* L., *A. cruentus* L. and *A. hypochondriacus* L.) in America. Plant Genet. Resour. Newsl.

[4] Costea M, DeMason DA (2001). Stem morphology and anatomy in *Amaranthus* L. (Amaranthaceae), taxonomic significance. J. Torrey Bot. Soc..

[5] Aderibigbe O (2022). Exploring the potentials of underutilized grain amaranth (*Amaranthus* spp.) along the value chain for food and nutrition security: A review. Crit. Rev. Food Sci. Nutr..

[6] Rastogi A, Shukla S (2013). Amaranth: A new millennium crop of nutraceutical values. Crit. Rev. Food Sci. Nutr..

[7] Mbwambo, O. I. *Morphological Characteristics, Growth and Yield of Elite Grain and Leaf Amaranth in Northern Tanzania*. Dissertation, Jomo Kenyatta University of Agriculture and Technology (2013).

[8] Martinez-Lopez A, Millan-Linares MC, Rodriguez-Martin NM, Millan F, Montserrat-de la Paz S (2020). Nutraceutical value of kiwicha (*Amaranthus caudatus L.*. Journal of Functional Foods..

[9] Ballabio C (2011). Biochemical and immunochemical characterization of different varieties of amaranth (*Amaranthus* L. ssp.) as a safe ingredient for gluten-free products. J. Agric. Food Chem..

[10] Usman MG, Rafii MY, Martini MY, Oladosu Y, Kashiani P (2017). Genotypic character relationship and phenotypic path coefficient analysis in chili pepper genotypes grown under tropical condition. J. Sci. Food Agric..

[11] Wattoo JI (2010). Study of correlation among yield related traits and path coefficient analysis in rice (*Oryza sativa* L.). Afr. J. Biotechnol..

[12] Binodh A, Manivannan N, Varman P (2008). Character association and path analysis in sunflower. Madras Agric. J..

[13] Tarekegne A (2001). Studies on Genotypic Variability and Inheritance of Waterlogging Tolerance in Wheat.

[14] Verty P, Prasad V, Collis J, Nazir M (2017). Correlation analysis in Gladiolus (*Gladiolus grandiflorus* L.). Agric. Res. Technol..

[15] Paredes-Lopez O (2018). Amaranth Biology, Chemistry, and Technology.

[16] Grubben G, Van Sloten D (1981). Genetic Resources of Amaranth—A Global Plan of Action.

[17] Shukla S (2006). Mineral profile and variability in vegetable amaranth (*Amaranthus tricolor*). Plant Foods Hum. Nutr..

[18] Grubben G, Van Sloten D (1981). Genetic Resources of Amaranths.

[19] Sari BG (2018). Interferência do tamanho de amostra no diagnóstico de multicolinearidade em análise de trilha. Pesquisa Agropecuária Bras..

[20] Cruz & Souza Carneiro (2006). Modelos Biométricos Aplicados ao Melhoramiento Genético.

[21] Figueiredo Neto A, Godinho M, Toth-Katona T, Palffy-Muhoray P (2005). Optical, magnetic and dielectric properties of non-liquid crystalline elastomers doped with magnetic colloids. Braz. J. Phys..

[22] Olivoto T (2017). Multicollinearity in path analysis: A simple method to reduce its effects. Agron. J..

[23] Viotto Carneiro PCS, Vilela de Resende MD, Lopes da Silva F, Peternelli LA (2020). Overcoming collinearity in path analysis of soybean [*Glycine max* (L.) Merr.] grain oil content. Plos one.

[24] Hair JF, Anderson RE, Tatham RL, Black WC (1995). Multivariate Date Analysis with Readings.

[25] Montgomery DC, Peck EA, Vining GG (2021). Introduction to Linear Regression Analysis.

[26] Singh & Chaudhary, B. D. *Biometrical Methods in Quantitative Genetic Analysis*. (1977).

[27] Dewey DR, Lu K (1959). A correlation and path-coefficient analysis of components of crested wheatgrass seed production 1. Agron. J..

[28] Wright, S. *Correlation and Causation*. (1921).

[29] Singh. *Biometrical Methods in Quantitative Genetic Analysis*. Vol. 318. Revised Ed. (Kalyani Pub. Ludhiana, 1985).

[30] Sravan T, Rangare N, Suresh B, Kumar SR (2012). Genetic variability and character association in rainfed upland rice (*Oryza sativa* L.). J. Rice Res..

[31] Hossain MB, Patras A, Barry-Ryan C, Martin-Diana AB, Brunton NP (2011). Application of principal component and hierarchical cluster analysis to classify different spices based on in vitro antioxidant activity and individual polyphenolic antioxidant compounds. J. Funct. Foods.

[32] Bayisa T, Amanuel M (2021). Estimate of correlation coefficients and path analysis for yield component traits in bread wheat (*Triticum aestivum* L.) genotypes under lowland temperature stress. Adv. Crop Sci. Tech..

[33] Sagar K, Hanchinamani C, Imamsaheb S, Nishani S, Ramanagouda S (2018). Genotypic correlation coefficients among growth, yield and quality parameters in Amaranthus genotypes (*Amaranthus tricolor* L.). Int. J. Curr. Microbiol. Appl. Sci..

[34] Jangde B, Asati BS, Sahu P, Tripathy B (2017). Correlation and path coefficient analysis in vegetable amaranthus (*Amaranthus tricolor* L.). J. Pharmacogn. Phytochem..

[35] Masum M (2020). Genetic Diversity, Correlation and Path Analysis in Stem Amaranth (Amaranthus lividus L.).

[36] Singh. Genetic variability diversity and stability analysis in grain amaranth *Amaranthus hypochondriacus* L. (2010).

[37] Olusanya AC (2018). A multi-species assessment of genetic variability in Nigerian Amaranthus accessions: Potential for improving intra-and interspecies hybridization breeding. Arch. Agron. Soil Sci..

[38] Akter N, Mian M, Islam M, Alim M, Islam M (2005). Estimation of genetic parameters, character association and path analysis in jute (*C. olitorius* L.) germplasm. Banglad. J. Plant Breed. Genet..

[39] Shrivastav SP, Yadav C, Singh V, Maurya V (2020). Evaluation and identification of most promising genotypes for varietal development in Amaranthus (*Amaranthus paniculatus* L.). Int. J. Curr. Microbiol. Appl. Sci..

[40] Tiwari J (2018). Variability and genetic parameters for different yield contributing traits in grain amaranth. Angrau.

[41] Gerrano, Jansen van Rensburg, W. & Adebola, P. *VI International Symposium on the Taxonomy of Cultivated Plants*. Vol. 1035. 183–187.

[42] Tejaswini N, Reddy KR, Saidaiah P, Ramesh T (2017). Correlation and path coefficient analysis in vegetable amaranth (*Amaranthus tricolor* L.) genotypes. Int. J. Curr. Microbiol. Appl. Sci..

[43] Salini K, Nirmalakumari A, Muthiah A, Senthil N (2010). Evaluation of proso millet (*Panicum miliaceum* L.) germplasm collections. Electron. J. Plant Breed..

[44] Sharma B, Sharma V (2013). Genetic variability, heritability and genetic advance studies in garden pea under mid hill conditions of Garhwal Himalaya. Environ. Ecol..

[45] Yadav R, Rana J, Ranjan J (2014). Analysis of variability parameters for morphological and agronomic traits in grain amaranth (*Amaranthus* sp) genotypes. Bioscan.

[46] Rani R, Mahato JL, Raj H (2019). Genetic analysis and inter-relationship of yield attributing traits in *Amaranthus* germplasm. J. Pharmacogn. Phytochem..

[47] Varalakshmi B, Pratap Reddy V (1997). Variability, heritability and correlation studies in vegetable amaranth. Indian J. Horticult..

[48] Kumar. *Collection, Evaluation and Identification of Suitable Genotypes of Amaranthus (Amaranthus* spp.*) for Chhattisgarh Plain Condition*. (Indira Gandhi Krishi Vishwavidyalaya Raipur, 2015).

[49] Olaniyi, A. S. *Morphology and Genetic Analysis of Vegetative Characterization of Four Grain Amaranth Accessions*. (2022).

[50] Akaneme F, Ani G (2013). Morphological assessment of genetic variability among accessions of *Amaranthus hybridus*. World Appl. Sci. J..

[51] Brenner, D. *et al. Genetic Resources and Breeding of Amaranthus*. Vol. 19. 227–285 (2000).

[52] Shukla (2018). Untapped amaranth (*Amaranthus* spp.) genetic diversity with potential for nutritional enhancement. Genet. Resour. Crop Evolut..

[53] Shayanowako AIT (2021). African leafy vegetables for improved human nutrition and food system resilience in southern Africa: A scoping review. Sustainability.

[54] Showemimo F, Soyombo MA, Amira JO, Porbeni JB (2021). Traits selection criteria for genetic improvement of grain and leafy Amaranth (*Amaranthus* spp) using principal component analysis. Egypt. J. Agric. Res..

[55] Garg N (2017). Genetic diversity in round gourd [*Praecitrullus fistulosus* (Stocks) Pangalo] accessions introduced from USDA for various qualitative and quantitative traits. J. Crop Improve..

[56] Onasanya R (2009). Growth and yield response of maize (*Zea mays* L.) to different rates of nitrogen and phosphorus fertilizers in southern Nigeria. World J. Agric. Sci..

[57] Nyasulu M, Sefasi A, Chimzinga S, Maliro M (2021). Agromophological characterisation of amaranth accessions from Malawi. Am. J. Plant Sci..

[58] Kumar. Studies on stability, correlation and path co-efficient analysis for grain yield and component traits in amaranth. *Glob. J. Agric. Res.* **6**, 22–37 (2018).

[59] Akin-Idowu PE, Gbadegesin MA, Orkpeh U, Ibitoye DO, Odunola OA (2016). Characterization of grain amaranth (*Amaranthus* spp.) germplasm in south west Nigeria using morphological, nutritional, and random amplified polymorphic DNA (RAPD) analysis. Resources.

[60] Aycicek M, Yildirim T (2006). Path coefficient analysis of yield and yield components in bread wheat (*Triticum aestivum* L.) genotypes. Pak. J. Bot..

[61] Shah LR, Afroza B, Khan S, Habib M (2018). Morphological characterization of *Amaranthus* spp. under temperate environment using NBPGR descriptor. J. Pharmacogn. Phytochem..

[62] Islam, M. S. *Genetic Diversity Analysis of Red Amaranth* (*Amaranthus cruentus *L.). (Department of Genetics and Plant Breeding, Sher-E-Bangla Agricultural, 2021).

[63] Alake CO, Otusanya GO (2020). Selection criteria for seed yield improvement in multi-species of *Amaranthus* genotypes. Int. J. Veg. Sci..

[64] Tomar, H., Kumar, V., Singh, S., Kumar, R. & Kumar, A. *Genetic Variability, Heritability, Correlation and Path Coefficient Analysis in Amaranth* (*Amaranthus spp.*) *in Western Uttar Pradesh* (*India*). (2022).

[65] Kumar & Yassin, M. Studies on stability, correlation and path co-efficient analysis for grain yield and component traits in amaranth. *Glob. J. Agric. Res.* **6**, 22–37 (2018).

[66] Sahu M, Tiwari A (2020). Genetic variability and association analysis of oat (*Avena sativa* L.) genotypes for green forage yield and other components. Curr. J. Appl. Sci. Technol..

[67] Wei F (2011). Rate and duration of grain filling of aerobic rice HD297 and their influence on grain yield under different growing conditions. ScienceAsia.

[68] Knott D, Gebeyehou G (1987). Relationships between the lengths of the vegetative and grain filling periods and agronomic characters in three durum wheat crosses 1. Crop Sci..

